# Vaccine Response in Patients With Multiple Sclerosis Receiving Teriflunomide

**DOI:** 10.3389/fneur.2022.828616

**Published:** 2022-02-28

**Authors:** Carlo Tornatore, Heinz Wiendl, Alex L. Lublin, Svend S. Geertsen, Jeffrey Chavin, Philippe Truffinet, Amit Bar-Or

**Affiliations:** ^1^Georgetown University Hospital, Washington, DC, United States; ^2^Department of Neurology With Institute of Translational Neurology, University of Münster, Münster, Germany; ^3^Sanofi, Cambridge, MA, United States; ^4^Sanofi, Chilly-Mazarin, France; ^5^Center for Neuroinflammation and Experimental Therapeutics and Department of Neurology, University of Pennsylvania, Philadelphia, PA, United States

**Keywords:** COVID-19, disease-modifying therapies (DMTs), multiple sclerosis, teriflunomide (Aubagio), vaccination

## Abstract

Many patients with multiple sclerosis (MS) receive disease-modifying therapies (DMTs), such as teriflunomide, to reduce disease activity and slow progression. DMTs mediate their efficacy by modulating or suppressing the immune system, which might affect a patient's response to vaccination. As vaccines against the SARS-CoV-2 virus become available, questions have arisen around their efficacy and safety for patients with MS who are receiving DMTs. Data are beginning to emerge regarding the potential influence of certain DMTs on a patient's response to coronavirus disease 2019 (COVID-19) vaccines and are supported by evidence from vaccination studies of other pathogens. This review summarizes the available data on the response to vaccines in patients with MS who are receiving DMTs, with a focus on teriflunomide. It also provides an overview of the leading COVID-19 vaccines and current guidance around COVID-19 vaccination for patients with MS. Though few vaccination studies have been done for this patient population, teriflunomide appears to have minimal influence on the response to seasonal influenza vaccine. The evidence for other DMTs (e.g., fingolimod, glatiramer acetate) is less consistent: some studies suggest no effect of DMTs on vaccine response, whereas others show reduced vaccine efficacy. No unexpected safety signals have emerged in any vaccine study. Current guidance for patients with MS is to continue DMTs during COVID-19 vaccination, though adjusted timing of dosing for some DMTs may improve the vaccine response.

## Introduction

Disease-modifying therapies (DMTs) for multiple sclerosis (MS) generally induce immunosuppression or immunomodulation by inhibiting expansion of stimulated lymphocytes (teriflunomide), redirecting pathological immune cells away from the central nervous system (natalizumab, sphingosine-phosphate receptor modulators), or depleting immune cell subsets (B and T cells; ocrelizumab, alemtuzumab, cladribine) (1, 2). These immunosuppressive and immunomodulatory modes of action introduce theoretical potential for increased infection risk, reduced vaccine effectiveness, or reduced duration of immunity (3–5). Whether these risks are real is beginning to be addressed in the 21st century, particularly regarding the seasonal influenza vaccine and certain DMTs such as interferon-beta (IFN-β) and teriflunomide.

The importance of understanding how DMTs might affect vaccination effectiveness and safety for patients with MS is highlighted amid the ongoing outbreak of coronavirus disease 2019 (COVID-19). As of October 2021, minimal data are available regarding potential influence of DMTs on the efficacy of vaccines for the SARS-CoV-2 virus. Most available data are for patients receiving ocrelizumab (6–9). Until substantial data have accumulated, evidence for other immunizations might provide insight into what can be expected with SARS-CoV-2 vaccines (4). This review summarizes currently available evidence on vaccine efficacy for patients receiving DMTs to treat MS, focusing on evidence for the DMT teriflunomide, and summarizes current guidance around SARS-CoV-2 vaccination in this population.

## How Vaccines Work

Entry of a pathogen into the body triggers the innate immune response, the first-line immune defense. The innate immune response engages rapidly but is not specifically directed against a particular pathogen. However, it triggers an adaptive immune response, which is pathogen specific and involves stimulation of T and/or B lymphocytes that may ultimately achieve highly focused immune memory against the pathogen (4, 10). For cell-mediated immunity, subsets of T lymphocytes are activated, and memory T cells are generated. For humoral immunity, B lymphocytes are stimulated to produce immunoglobulin M (IgM) and then IgG antibodies against the pathogen. The antibodies and memory cells generated in response to the pathogen generally persist in the body after the pathogen is eliminated, maintaining immunological memory (10).

Several vaccine types have been developed: earlier vaccines used live-attenuated or inactivated pathogens to induce immunity, whereas modern vaccines may be based on toxoids or parts of pathogens, such as viral vectored vaccines and those based on mRNA, DNA, virus-like particles, or recombinant protein (10, 11). Regardless of the type, nearly all vaccines work by introducing antigen, or genes that encode antigen, in the immune system (11, 12). An adjuvant is often included to stimulate the innate immune system to recognize pathogen-associated molecular patterns, but some modern vaccines use these patterns instead of adjuvants. The antigen directs the adaptive immune response against a specific pathogen (11). While both the humoral and cellular responses generated by vaccines can contribute to protection from pathogens, vaccine response is generally measured according to IgG antibody titers raised against the particular antigen, as measuring antibody responses is generally easier than measuring cell-based responses (4, 11).

## Vaccination Protocols in Patients with MS Receiving DMTs (Pre-COVID-19)

### Teriflunomide

Two studies have investigated whether teriflunomide affects vaccine response (Table 1). One study evaluated the immune response to the 2011/2012 influenza vaccine (containing H1N1, H3N2, and B strains) in 128 MS patients receiving teriflunomide 7 or 14 mg or IFN-β-1a (13). In that study, ≥90% of patients in all treatment groups achieved seroprotection against the H1N1 and B strains, with seroprotection defined, in accordance with European guidelines for evaluating vaccine response (29), as post-vaccination antibody titers ≥40. For the H3N2 strain, seroprotection rates were also ≥90% with teriflunomide 7 mg and IFN-β-1a, but were 76.9% with teriflunomide 14 mg. The 2011/2012 influenza vaccine included the same strains as were used in the 2010/2011 vaccine, which had been previously administered to 44–70% of study participants. As a result, many patients had H1N1, H3N2, and B strain antibody titers ≥40 at baseline. Of those with pre-vaccination antibody titers <40, >61% of patients in the teriflunomide 7 or 14 mg groups and ≥81% of patients in the IFN-β-1a group achieved seroprotection with the 2011/2012 vaccine, with the highest seroconversion rates observed for the B (teriflunomide 7 mg, 91.7% seroconversion; teriflunomide 14 mg, 91.7%; IFN-β-1a, 85.0%) and H1N1 strains (85.7, 80.0, and 80.0%, respectively) and lower seroconversion rates for the H3N2 strain (77.8, 61.1, and 81.8%, respectively) (13). The results of this study suggest teriflunomide generally does not inhibit a patient's ability to mount an effective immune response to recall antigens with the influenza vaccine.

**Table 1:** Assessed responses to selected vaccines in patients receiving DMTs.

**DMT**	**Study design**	**Groups compared**	**Vaccine type**	**Assessment of immune response**	**Key findings**	**References**
Teriflunomide	TERIVA Open-label, parallel-group (*N* = 128)	Patients receiving teriflunomide 7 or 14 mg vs. IFN-β-1	Influenza	Antibody titers ≥40 at 28 days after vaccination	Post-vaccination titers ≥40 achieved for ≥90% of patients in all groups (H1N1) and ≥90% of patients receiving teriflunomide 7 mg or IFN-β-1 (H3N2; 77% response with teriflunomide 14 mg)	(13)
	Randomized, double-blind, placebo-controlled (*N* = 46)	Healthy controls receiving teriflunomide 14 mg vs. placebo	Rabies	Antibody titers (>0.5 IU/mL)	Teriflunomide did not limit the ability to achieve seroprotective titers against neoantigen. However, antibody titers were lower with teriflunomide than with placebo	(14)
IFN-β	Prospective, non-randomized, open-label (*N* = 163)	Patients receiving IFN-β-1a vs. patients not receiving IFN-β-1a	Influenza	HI titers (≥40 indicated seroprotection)	Similar proportions of patients achieved seroprotection IFN-β-1a, 93.0%; no IFN-β-1a, 90.9%)	(15)
	Open-label, observational, retrospective-prospective (*N* = 59)	Patients receiving IFN-β vs. healthy controls	Influenza	Influenza-specific T cells; anti-influenza A and B IgM and IgG titers	Influenza-specific T cell frequencies and IgG titers increased similarly in both groups, indicating a strong immune response	(16)
IFN-β, DMF	Open-label (*N* = 71)	DMF vs. IFN	Tetanus, diphtheria	Proportion of patients with ≥2-fold increase in antitoxoid titers by 4 weeks after vaccination	Response rates were similar for DMF vs. IFN: tetanus, 68 vs. 73%; diphtheria, 58 vs. 61%	(17)
			Meningococcal		Response rate was 53% for both groups	
			Pneumococcal		Response rates were similar for DMF vs. IFN: pneumococcal serotype 3, 66 vs. 79%; pneumococcal serotype 8, 95 vs. 88%	
Fingolimod	Prospective, observational, open-label (*N* = 32)	Patients receiving fingolimod vs. healthy controls	Influenza	Lymphocyte counts; frequency of influenza-specific cells; virus-specific T cell responses	Lymphocyte counts decreased 64% vs. the lower limit of normal for patients receiving fingolimod Similar frequencies of influenza-specific IFN-γ-secreting T cells were observed between patients receiving fingolimod and healthy controls Patients achieved a normal T cell response to vaccine despite S1PR blockade with fingolimod	(18)
Fingolimod	Blinded, randomized, placebo-controlled (*N* = 136)	Patients receiving fingolimod vs. placebo	Influenza	Proportion of patients achieving seroconversion or ≥4-fold increase in antibody titers against ≥1 influenza strain or seroconversion against tetanus vaccine	Response rates were 54% (fingolimod) vs. 85% (placebo) at 3 weeks and 43 vs. 75% at 6 weeks post vaccination	(19)
			Tetanus		Response rates were reduced for fingolimod vs. placebo at 3 weeks (40 vs. 61%) and 6 weeks (38 vs. 49%) after vaccination	
Natalizumab	Randomized, open-label (*N* = 60)	Patients receiving natalizumab vs. untreated controls	Tetanus, KLH	Adequate response, defined as ≥2-fold increase in specific serum IgG 28 days after vaccination	All evaluable patients had adequate response to tetanus toxoid; the proportions of responders to tetanus and KLH vaccines were similar with vs. without natalizumab	(20)
Ocrelizumab	VELOCE/ Phase 3b, open-label (*N* = 102)	Patients receiving ocrelizumab vs. controls (IFN-β or no DMT)	Influenza	Hemagglutination inhibition titers (≥40 indicated seroprotection)	Seroprotection was achieved by 55.6–80.0% of patients receiving ocrelizumab vs. 97.0% of controls	(21)
			Tetanus, KLH	Proportion of patients with a positive response 8 weeks after vaccination (anti-TT IgG antibody titer ≥0.2 IU/mL)	Response rates were reduced with ocrelizumab vs. controls to tetanus (23.9 vs. 54.5%) and Pneumovax (71.6 vs. 100%) vaccines; humoral response to KLH was reduced with ocrelizumab	
			Pneumococcal (13-PCV, 23-PPV)	Proportion of patients with a positive response 4 weeks after vaccination (≥2-fold increase in IgG titers)	Response rates to 23-PPV were reduced with ocrelizumab (71.6%) vs. controls (100%)	
Alemtuzumab	Pilot, historical case-control (*N* = 23)		Influenza	Rates of seroprotection (≥2-fold increase in antibodies)	100% of patients who received the influenza vaccine achieved seroprotection; 95% achieved ≥4-fold increase in antibody titers, compared with 82–90% of historical controls	(22)
	(*N* = 22)		Diphtheria, tetanus, polio-myelitis		Post-vaccine rates of seroprotection were 95–100% for patients receiving alemtuzumab	
	(*N* = 23)		Meningococcal group C		91% of patients achieved seroprotection vs. 97.6–100% of historical controls	
	(*N* = 21)		Pneumococcal (23-PPV)		Serotype 3: 73% of patients achieved seroconversion vs. 35–47% of historical controls Serotype 8: 95% of patients achieved seroconversion vs. 81–85% of historical controls	
Daclizumab-β	SELECTED Open-label, single-arm, prospective (*N* = 90)	Daclizumab-β	Influenza	Hemagglutination inhibition titers (≥40 indicated seroprotection)	Seroprotection achieved for 92% (strain A/H1N1), 91% (A/H3N2), and 67% (B) of patients	(23)
Multiple	Prospective, non-randomized, observational (*N* = 108)	Patients receiving IFNs, glatiramer acetate, natalizumab, fingolimod, or other DMTs	Influenza	Proportion of patients achieving seroconversion or seroprotection; mean geometric titer increase; proportion of patients achieving HI titer ≥40	Rates of seroprotection were highest in H1N1 strain (71.4–100%), compared with H3N2 (28.6–33.3%) or B strains (57.1–88.9%) Overall seroprotection was highest in patients receiving IFNs (73.3%) and glatiramer acetate (57.7%) and lowest in those receiving natalizumab (14.3%) and fingolimod (33.3%)	(24)
Multiple	Prospective, non-randomized, open-label (*N* = 152)	Patients receiving fingolimod, glatiramer acetate, IFN-β-1a/b, natalizumab, or no DMT vs. healthy controls	Influenza	Seroprotection rates at 3, 6, and 12 months	At 3–12 months post vaccination, seroprotection rates were reduced for patients receiving natalizumab (55.6–75.0% protected) or fingolimod (22.2–71.4%) vs. healthy controls (70.4–94.6%); patients receiving glatiramer acetate (77.3–91.3%) or IFN-β-1a/b (79.0–88.0%) achieved similar protection to controls	(25)
Multiple	Observational, prospective (*N* = 329)	Patients receiving IFN-β, glatiramer acetate, natalizumab, or mitoxantrone vs. controls	Influenza	Seroprotection rates (defined as HI ≥ 40)	Rates of seroprotection varied by DMT and influenza strain (H1N1, H3N2); patients receiving IFN-β had similar response rates as healthy controls, whereas rates were generally lower with glatiramer acetate, natalizumab (except for 2010 H1N1 strain), and mitoxantrone	(26)
Multiple	Observational multicenter prospective cohort (*N* = 780)	Patients with MS receiving ocrelizumab, natalizumab, fingolimod, IFNs, teriflunomide, other DMTs, or untreated	SARS-CoV-2	SARS-CoV-2 antibodies before 1st vaccination and 4 weeks after 2nd vaccination	Compared with untreated patients, post-vaccination antibody levels were significantly lower for patients receiving ocrelizumab (201-fold reduction), fingolimod (26-fold reduction), or rituximab (20-fold) (*P* < 0.001 for all). No significant difference was observed with teriflunomide or other DMTs	(27)
Multiple	Observational cohort (*N* = 172)	Patients with MS receiving DMTs vs. healthy controls		SARS-CoV-2 IgG antibody titers	Sufficient antibody response to vaccination was observed for 97.9–100% of healthy controls, untreated MS patients, and patients receiving cladribine, but for only 3.8% receiving fingolimod and 22.7% receiving ocrelizumab	(7)
Multiple	Observational cohort (*N* = 120)	Patients with MS receiving DMTs		SARS-CoV-2 IgG antibody titers	Compared with patients receiving no MS therapy, the IgG antibody response to vaccination was reduced for those receiving anti-CD20 antibodies (β = −2.19; *P* < 0.001) or sphingosine-1-phosphate modulators (β = −1.92; *P* < 0.001); no difference was observed for patients receiving teriflunomide (β = −0.01; *P* = 0.98) or cladribine (β = −0.39; *P* = 0.44)	(28)

*13-PCV, 13-valent pneumococcal conjugate; 23-PPV, 23-valent pneumococcal polysaccharide; DMF, dimethyl fumarate; DMT, disease-modifying therapy; HI, hemagglutination inhibition; IFN, interferon; Ig, immunoglobulin; KLH, keyhole limpet hemocyanin; MS, multiple sclerosis; S1PR, sphingosine-1-phosphate inhibitor*.

The second study evaluated the immune response to neoantigen and recall antigens with the human diploid cell rabies vaccine (HDCV) in healthy volunteers who received teriflunomide or placebo for 30 days (14). No participants had measurable antibodies to the rabies virus before vaccination, and all achieved seroprotection against the neoantigen, defined as antibody titers ≥0.5 IU/mL, within 1 week after second vaccination. By days 31 and 38 (2 and 3 weeks after second vaccination), geometric mean antibody titers were significantly lower with teriflunomide (7.8 and 15.2 IU/mL) vs. placebo (12.5 and 28.9 IU/mL), but remained well-above the 0.5 IU/mL threshold for all participants, indicating sustained seroprotection against the rabies neoantigen.

To assess cellular recall (memory) responses, the second study also evaluated delayed-type hypersensitivity (DTH) responses to *Candida albicans, Trichophyton*, and tuberculin purified protein derivative recall antigens (14). For DTH assessments, positive DTH reaction indicated an appropriate cellular memory response to the recall antigen. In both the teriflunomide and placebo groups, numbers of participants with positive DTH responses to *C. albicans* and *Trichophyton* antigens declined from screening to day 30, but proportions of patients with a positive response to tuberculin purified protein derivative antigen were unchanged. Moreover, no differences were observed between treatment groups for any recall antigen, suggesting teriflunomide does not influence the cellular memory response to recall antigens.

### Interferons

Some studies with the seasonal influenza vaccine have linked IFN-β with high rates of seroprotection, similar to those in healthy controls (13, 15, 16, 24–26). In an early study, 93% of patients receiving IFN-β-1a and 91% of patients receiving no DMT achieved seroprotection with the influenza vaccine, and no MS relapses occurred during the month after vaccination (15). More recent studies of patients receiving IFN-β have reported increased frequencies of influenza-specific T cells and antibody titers after vaccination (16) and seroprotection rates comparable to or higher than those of healthy controls (−0.9 to 17.0% difference), with seroprotection maintained up to 12 months after vaccination (25, 26). A higher proportion of patients receiving IFN-β achieved seroprotection against multiple influenza strains compared with patients receiving glatiramer acetate, natalizumab, or mitoxantrone (26). Adverse events of flu-like symptoms, headache, and weakness have been reported after influenza vaccination for patients receiving IFN treatment (24).

IFN-β and dimethyl fumarate were associated with similar rates of protection, defined as ≥2-fold increase in IgG antibody titers, against tetanus antitoxoid (73 and 68%, respectively), diphtheria antitoxoid (61 and 58%), pneumococcal vaccine polyvalent serotypes 3 (79 and 66%) and 8 (88 and 95%), and meningococcal group C (53 and 53%) (17). Severe adverse events of injection-site cellulitis, burning sensation, and arthralgia were reported for two patients in the IFN-β group (*n* = 33) and none in the dimethyl fumarate group (*n* = 38) (17).

### Glatiramer Acetate

In one study, patients who received glatiramer acetate achieved similar rates of protection with the influenza vaccine vs. healthy controls (−7.6 to 6.9% difference over 12 months) (25). However, a separate study reported seroprotection rates that were lower (12.9–37.8%) for patients receiving glatiramer acetate than for healthy controls (26). Of note, seroprotection may vary according to influenza strain for patients receiving glatiramer acetate, fingolimod, or natalizumab, with higher rates of protection against strain H1N1 than against strains H3N2 or B (24, 26). Flu-like symptoms, increased temperature, and nightly sweating have been reported after influenza vaccination for patients receiving glatiramer acetate (24).

### Sphingosine-1-Phosphate Inhibitors

One small study of fingolimod found no reduction in humoral or cellular response to the influenza vaccine (18), but two other studies reported significantly reduced rates of seroprotection with fingolimod vs. controls (23.2–48.2% difference over 12 months) (19, 25). Exanthema has been reported after influenza vaccination in some patients receiving fingolimod (24).

One study suggested that patients receiving fingolimod might have reduced protection against tetanus toxoid boosters compared with patients receiving placebo (6-week responder rates, 38 vs. 49%, respectively) (19).

### Monoclonal Antibodies

Natalizumab appears to diminish the response to seasonal influenza vaccines compared with healthy controls (26) or patients receiving IFN-β (25). Natalizumab might also attenuate response to the tetanus vaccine and keyhole limpet hemocyanin (KLH); although overall response rates were similar between patients taking natalizumab and those who delayed natalizumab treatment, a trend toward lower IgG antibody titers was observed for patients receiving natalizumab (20).

In one study, an attenuated response to influenza vaccination was observed in patients receiving ocrelizumab (55.6–80.0% seroprotection) vs. controls (patients receiving IFN-β therapy or no DMT; 75.0–97.0%) (21). Ocrelizumab was also associated with lower response rates to tetanus (−30.7% difference in 8-week response rate), pneumococcal (−65.3 to −19.1% difference in 8-week response rates), and KLH vaccines (3.0- and 6.1-fold difference in IgM and IgG titers at 8 weeks, respectively) (21). Safety findings with ocrelizumab were consistent with the safety profile from phase 3 studies and most commonly involved mild or moderate infections (47%), which occurred more frequently than in controls (15%) (21).

Alemtuzumab may not interfere with responses to T cell–independent recall antigen (pneumococcus) or T cell–dependent recall antigen (tetanus, diphtheria, polio) and neoantigen (meningococcus C), although vaccination within 6 months of treatment resulted in a smaller proportion of responders (22). However, the results should be interpreted with caution, as they are based on a single study that compared a small population (*N* = 24) with literature controls.

## COVID-19 Vaccination for Patients with MS

Like many pathogens, SARS-CoV-2 initially infects mucosal surfaces of the upper respiratory and digestive tracts (30). Mucosal surfaces use adaptive immune mechanisms for protection that involve secretory IgA and differ from those of other epithelial layers or at a systemic level (11). The mucosal immune response is non-inflammatory, but an inflammatory immune response dominates once a pathogen reaches terminal airways and alveoli (30).

To enter target cells, the SARS-CoV-2 virus uses a spike glycoprotein that binds the ACE2 receptor (31). Because the spike protein is essential for entry into the target cell, all vaccines use this protein as the immunizing antigen (32). The intent is to present the spike protein to the immune system, generate neutralizing antibodies that prevent it binding the ACE2 receptor, and thereby limit viral entry and infection of host cells (31, 32). As of November 2021, the COVID-19 vaccine developed by Pfizer-BioNTech has received full approval from the US FDA for use in adults (≥16 years) and is authorized for emergency use in children (5–15 years); the Moderna and Johnson & Johnson/Janssen are authorized for emergency use by the US FDA in adults (≥18 years). These vaccines are recommended by the CDC to prevent SARS-CoV-2 infection and COVID-related complications (Table 2).^1^ Additional vaccines from AstraZeneca, Pfizer-BioNTech, Novavax, Inovio, and Medicago are also in Phase 3 development in the US (Table 2). This short list comprises a variety of vaccine types: both vaccines from Pfizer-BioNTech (BNT162b1 and BNT162b2) and the vaccine from Moderna (mRNA-1273) are nucleic acid (mRNA) based (33, 34); the Inovio vaccine is DNA based^2^; the Janssen and AstraZeneca vaccines are viral vector vaccines, which consists of inactivated adenovirus (35, 36); the Novavax vaccine is protein based and contains spike glycoproteins (37); and the Medicago vaccine uses a plant-based coronavirus-like particle.^3^ In other countries, a recombinant adenovirus–based vaccine developed by Russian researchers and inactivated SARS-CoV-2 vaccines developed by Sinovac and Sinopharm are demonstrating favorable efficacy against SARS-CoV-2 infection, but none is in Phase 3 development in the US (38–40). None of the vaccines currently available or in Phase 3 development in the US contain replication-competent virus. Only one vaccine, from Meissa Vaccines, uses live attenuated virus and is in phase 1 clinical trials in the US.

**Table 2:** COVID-19 vaccines authorized for emergency use^†^ or in phase 3 development in the US as of October 20, 2021.

**Vaccine type**	**Name**	**Primary developers**	**Country of origin**	**Date of approval or emergency use authorization in the US and EU**
mRNA based	BNT162b2	Pfizer-BioNTech	Multinational	US: Approval August 23, 2021 EU: December 21, 2020
mRNA based	BNT162b1	Pfizer-BioNTech	Multinational	US: N/A (Phase 3 clinical trials) EU: N/A (Phase 2 clinical trials)
mRNA based	mRNA-1273	Moderna	US	US: EUA December 18, 2020 EU: January 6, 2021
DNA based	INO-4800	Inovio	US	US: N/A (Phase 3 clinical trials) EU: N/A
Adenovirus	AZD1222	AstraZeneca	UK	US: N/A (Phase 3 clinical trials) EU: January 29, 2021
Non-replicating viral vector	JNJ-78436735 (Ad26.COV2.S)	Johnson & Johnson/Janssen	US	US: February 27, 2021 EU: March 11, 2021
Nanoparticle	NVX-CoV2373	Novavax	US, Mexico	US: N/A (Phase 3 clinical trials) EU: December 20, 2021
Plant-based virus-like particle	CoVLP	Medicago	US, Canada	US: N/A (Phase 3 clinical trials) EU: N/A

*EUA, Emergency use authorization; N/A, not applicable*.

† *https://www.cdc.gov/coronavirus/2019-ncov/vaccines/different-vaccines.html*.

Though guidance has been issued for patients with MS who are considering vaccination against the SARS-CoV-2 virus, the information is based largely on studies using other vaccines, most of which used inactivated or toxoid- or polysaccharide-based vaccines (4, 11). Initial data are emerging on the interaction between DMTs and the development of SARS-CoV-2 antibodies. A single-center study (*N* = 555) that included untreated patients and patients receiving DMTs suggested the rates of adverse events, reported a median 38 days after the first BNT162b2 dose and 20 days after the second dose, were similar to those in the general population: 29.7% after first dose, and 40.2% after second dose (6). Of the 125 patients from that study for whom seroprotection was assessed 1 month after the second dose, humoral immunity was attained for 22.7% of patients receiving ocrelizumab, 3.8% receiving fingolimod, 100% receiving cladribine, and 100% of untreated patients (7). A separate study also reported reduced odds of antibody formation after SARS-CoV-2 infection with ocrelizumab vs. other DMTs (odds ratio, 0.045; *P* = 0.011) (41). A multicenter cohort study of patients with MS reported that compared with patients receiving no DMT, post-vaccination antibody levels 1 month after the second dose of the BNT162b2 or mRNA-1273 vaccine were significantly (*P* < 0.001) lower in patients receiving ocrelizumab (201-fold reduction), fingolimod (26-fold), and rituximab (20-fold) compared with untreated patients (27). Patients receiving teriflunomide had similar antibody levels to untreated patients. Across cohorts, antibody levels were 3.5-fold higher for patients who received the mRNA-1273 vs. BNT162b2 vaccine. Similar findings were reported in a Swiss cohort of patients with MS who received the BNT162b2 or mRNA-1273 vaccine (28). Compared with patients who received no MS therapy, SARS-CoV-2 IgG titers 21–35 days after the second dose were lower in patients receiving anti-CD20 antibodies or sphingosine-1-phosphate modulators; no significant difference was found with teriflunomide or cladribine. Of note, although patients with MS who are treated with an anti-CD20 antibody have poor antibody responses to SARS-CoV-2 vaccination, these patients appear to have a robust cellular response by 1 month after the second vaccine dose, which suggests that some level of immunity is attained (42). A robust cellular response to SARS-CoV-2 vaccination was also recently observed in the majority of patients on teriflunomide and most other DMTs except sphingosine-1-phosphate modulators (43, 44).

Based on accumulated evidence for vaccination in the context of MS DMTs, an international consensus panel, as well as the US National MS Society (NMSS) and UK MS Society (MSS), consider the potential benefit of COVID-19 vaccination to outweigh the risk of avoiding vaccination, with regard to both individual and public safety (32).^4^,^5^ All recommendations suggest the vaccines are safe for patients with MS, including those receiving DMTs, and are unlikely to cause MS relapse, worsen MS symptoms, or reduce DMT efficacy. The NMSS emphasizes the importance of vaccination for high-risk populations with MS. These include black and Hispanic patients and patients with progressive MS, who are older, or who have greater physical disability or medical conditions that might increase the severity of COVID-19 symptoms.

According to these guidance statements, as of September 2021, patients with MS do not need to postpone initiating most DMTs or adjust the timing of administration. Whereas, the consensus statement from Centonze et al. recommends not discontinuing or modifying DMTs to improve vaccine efficacy (32), the NMSS and MSS note exceptions for certain DMTs. In the NMSS guidance, exceptions include patients initiating treatment with fingolimod, siponimod, ozanimod, alemtuzumab, cladribine, ocrelizumab, rituximab, and ofatumumab. Ideally these DMTs would not be initiated until 2–4 weeks after the second vaccine injection. The MSS guidance similarly recommends patients wait until after vaccination before initiating treatment with ocrelizumab or rituximab. For patients already receiving alemtuzumab, ocrelizumab, rituximab, and ofatumumab, the NMSS suggests the first injection of COVID-19 vaccine be administered ≥12 weeks (≥4 weeks for ofatumumab) after the last DMT dose, with the DMT resumed ≥4 weeks after the second injection. The MSS echoes this guidance for patients already receiving alemtuzumab and cladribine. In addition, patients receiving high-dose steroids should wait at least 3–5 days after the last steroid dose before receiving a vaccine injection.

Neither society has provided guidance around COVID-19 vaccination for patients experiencing MS relapse or exacerbation. However, AAN guidelines recommend such patients postpone any vaccinations until their relapse is no longer active (45).

## Discussion

During the first year of the COVID-19 pandemic, several prescribers and their patients with MS questioned whether DMTs might increase the risk for infection, and potentially lead to severe COVID-19 symptoms (46). Early evidence on patients receiving MS DMTs at the time of a COVID-19 diagnosis suggests that despite their immunomodulatory or immunosuppressive mechanisms of action, most DMTs, including teriflunomide, do not increase the risk of more severe COVID-19 symptoms (3). Among the available literature, most notable are a systematic review of 2,493 patients with MS who were infected with SARS-CoV-2, of whom 2,047 were receiving DMTs (47), and a description of 873 patients with MS and SARS-CoV-2 infection, 761 of whom were receiving DMTs (48). Both publications suggest most DMTs do not increase the severity of COVID-19 symptoms or contribute to poorer outcomes; the risk of serious outcomes may be higher for untreated patients or those receiving anti-CD20 therapies (47). It is possible that some DMTs might attenuate the immune response to the SARS-CoV-2 virus in patients with MS. For example, some investigators have hypothesized that the strong antigenic stimulation often caused by SARS-CoV-2 may be moderated by teriflunomide, which reduces the level of immune activation without inducing major immunosuppression (49–51). Others have proposed that teriflunomide might harbor antiviral properties that could reduce the ability of the virus to replicate in the infected cell (47, 52). Because limited evidence is available to date, it is not yet possible to draw conclusions about the effects of teriflunomide or any other DMT on the course of SARS-CoV-2 infection. However, the NMSS suggests the benefits of continuing MS treatments outweigh the potential risks of stopping treatment.^6^

In patients with MS, use of DMTs may complicate the evidence around vaccinations due to their immunomodulatory and immunosuppressive effects (4). Initial evidence suggests the^6^ possibility that some DMTs might reduce efficacy of vaccines, including vaccines for the SARS-CoV-2 virus (7, 27). The available evidence is encouraging, as it suggests that compared with healthy controls, patients with MS receiving most DMTs, including teriflunomide, do not have reduced response to vaccines or experience adverse effects from vaccination.

## Author Contributions

CT, HW, AL, SG, JC, PT, and AB-O provided substantial contributions to the interpretation of data for the work, provided critical revision of drafts, approved the manuscript for submission, and agreed to be accountable for all aspects of the work. All authors contributed to the article and approved the submitted version.

## Funding

Editorial support was funded by Sanofi.

## Conflict of Interest

CT reports receiving research support and honoraria for speaking or acting as a member of an advisory board or a Data and Safety Monitoring Board for Atara, Biogen, Celgene, Genentech, Sanofi Genzyme, and TG Therapeutics. HW reports receiving honoraria for acting as a member of scientific advisory boards for Biogen, Genzyme, Merck Serono, Novartis, Roche Pharma AG, Sanofi-Aventis, and UCB; speaker honoraria and travel support from Alexion, Biogen, Biologix, Cognomed, F. Hoffmann-La Roche Ltd., Gemeinnützige Hertie-Stiftung, Merck, Novartis, Roche Pharma AG, Sanofi Genzyme, TEVA, and WebMD Global; consulting fees from Actelion, Argenx, Biogen, Bristol Myers Squibb, EMD Serono, Idorsia, IGES, Immunic, Immunovant, Janssen, Johnson & Johnson, Novartis, Roche, Sanofi, the Swiss Multiple Sclerosis Society, and UCB; and research funding from the German Ministry for Education and Research (BMBF), Deutsche Forschungsgemeinschaft (DFG), Else Kröner Fresenius Foundation, Fresenius Foundation, the European Union, Hertie Foundation, NRW Ministry of Education and Research, Interdisciplinary Center for Clinical Studies (IZKF) Muenster and Biogen, GlaxoSmithKline, Roche Pharma AG, and Sanofi Genzyme. AL, SG, JC, and PT report receiving compensation as employees of Sanofi, and may hold shares and/or stock options in the company. AB-O reports receiving consulting fees from Accure, Atara Biotherapeutics, Biogen, BMS/Celgene/Receptos, GlaxoSmithKline, Gossamer, Janssen/Actelion, Medimmune, Merck/EMD Serono, Novartis, Roche/Genentech, and Sanofi Genzyme; and has received grant support to the University of Pennsylvania from Biogen Idec, Roche/Genentech, Merck/EMD Serono and Novartis.

## Publisher's Note

All claims expressed in this article are solely those of the authors and do not necessarily represent those of their affiliated organizations, or those of the publisher, the editors and the reviewers. Any product that may be evaluated in this article, or claim that may be made by its manufacturer, is not guaranteed or endorsed by the publisher.

## Footnotes

^1^https://www.cdc.gov/coronavirus/2019-ncov/vaccines/different-vaccines.html.

^2^https://www.inovio.com/our-focus-serving-patients/covid-19/.

^3^https://www.medicago.com/en/covid-19-programs/.

^4^https://www.mssociety.org.uk/what-we-do/news/ms-society-medical-advisers-release-consensus-statement-covid-19-vaccines.

^5^https://www.nationalmssociety.org/coronavirus-covid-19-information/multiple-sclerosis-and-coronavirus/covid-19-vaccine-guidance.

^6^https://www.nationalmssociety.org/coronavirus-covid-19-information/multiple-sclerosis-and-coronavirus/ms-treatment-guidelines-during-coronavirus.

## Abbreviations

COVID-19: coronavirus disease 2019
DMT: disease-modifying therapy
DTH: delayed-type hypersensitivity
IFN-β: interferon-beta
Ig: immunoglobulin
KLH: keyhole limpet hemocyanin
MS: multiple sclerosis
MSS: MS Society
NMSS: National MS Society.

## References

[1] Jakimovski D Weinstock-Guttman B Ramanathan M Dwyer MG Zivadinov R. Infections, vaccines and autoimmunity: a multiple sclerosis perspective. Vaccines. (2020) 8:50. 10.3390/vaccines8010050 32012815 PMC7157658

[2] McGinley MP Goldschmidt CH Rae-Grant AD. Diagnosis and treatment of multiple sclerosis: a review. JAMA. (2021) 325:765–79. eng. 10.1001/jama.2020.26858 33620411

[3] Zheng C Kar I Chen CK Sau C Woodson S Serra A. Multiple sclerosis disease-modifying therapy and the COVID-19 pandemic: implications on the risk of infection and future vaccination. CNS Drugs. (2020) 34:879–96. 10.1007/s40263-020-00756-y 32780300 PMC7417850

[4] Ciotti JR Valtcheva MV Cross AH. Effects of MS disease-modifying therapies on responses to vaccinations: a review. Mult Scler Relat Disord. (2020) 45:102439. 10.1016/j.msard.2020.102439 32769063 PMC7395588

[5] Williamson EM Chahin S Berger JR. Vaccines in multiple sclerosis. Curr Neurol Neurosci Rep. (2016) 16:36. 10.1007/s11910-016-0637-6 26922172

[6] Achiron A Dolev M Menascu S Zohar DN Dreyer-Alster S Miron S. COVID-19 vaccination in patients with multiple sclerosis: what we have learnt by February 2021. Mult Scler. (2021) 27:864–70. 10.1177/13524585211003476 33856242 PMC8114441

[7] Achiron A Mandel M Dreyer-Alster S Harari G Magalashvili D Sonis P. Humoral immune response to COVID-19 mRNA vaccine in patients with multiple sclerosis treated with high-efficacy disease-modifying therapies. Ther Adv Neurol Disord. (2021) 14:17562864211012835. 10.1177/17562864211012835 34035836 PMC8072850

[8] Buttari F Bruno A Dolcetti E Azzolini F Bellantonio P Centonze D. COVID-19 vaccines in multiple sclerosis treated with cladribine or ocrelizumab. Mult Scler Relat Disord. (2021) 52:102983. 10.1016/j.msard.2021.102983 33990054 PMC8093161

[9] Gallo A Capuano R Donnarumma G Bisecco A Grimaldi E Conte M. Preliminary evidence of blunted humoral response to SARS-CoV-2 mRNA vaccine in multiple sclerosis patients treated with ocrelizumab. Neurol Sci. (2021) 42:3523–6. 10.1016/j.jns.2021.117795 34128150 PMC8203306

[10] Vetter V Denizer G Friedland LR Krishnan J Shapiro M. Understanding modern-day vaccines: what you need to know. Ann Med. (2018) 50:110–20. 10.1080/07853890.2017.1407035 29172780

[11] Iwasaki A Omer SB. Why and how vaccines work. Cell. (2020) 183:290–5. 10.1016/j.cell.2020.09.040 33064982 PMC7560117

[12] Mendonca SA Lorincz R Boucher P Curiel DT. Adenoviral vector vaccine platforms in the SARS-CoV-2 pandemic. NPJ Vaccines. (2021) 6:97. 10.1038/s41541-021-00356-x 34354082 PMC8342436

[13] Bar-Or A Freedman MS Kremenchutzky M Menguy-Vacheron F Bauer D Jodl S. Teriflunomide effect on immune response to influenza vaccine in patients with multiple sclerosis. Neurology. (2013) 81:552–8. 10.1212/WNL.0b013e31829e6fbf 23851964 PMC3744268

[14] Bar-Or A Wiendl H Miller B Benamor M Truffinet P Church M. Randomized study of teriflunomide effects on immune responses to neoantigen and recall antigens. Neurol Neuroimmunol Neuroinflamm. (2015) 2:e70. 10.1212/NXI.0000000000000070 25738167 PMC4335822

[15] Schwid SR Decker MD Lopez-Bresnahan M Rebif-Influenza Vaccine Study I. Immune response to influenza vaccine is maintained in patients with multiple sclerosis receiving interferon beta-1a. Neurology. (2005) 65:1964–6. 10.1212/01.wnl.0000188901.12700.e0 16380621

[16] Mehling M Fritz S Hafner P Eichin D Yonekawa T Klimkait T. Preserved antigen-specific immune response in patients with multiple sclerosis responding to IFNbeta-therapy. PLoS ONE. (2013) 8:e78532. 10.1371/journal.pone.0078532 24223820 PMC3818403

[17] von Hehn C Howard J Liu S Meka V Pultz J Mehta D. Immune response to vaccines is maintained in patients treated with dimethyl fumarate. Neurol Neuroimmunol Neuroinflamm. (2018) 5:e409. 10.1212/NXI.0000000000000409 29159204 PMC5688262

[18] Mehling M Hilbert P Fritz S Durovic B Eichin D Gasser O. Antigen-specific adaptive immune responses in fingolimod-treated multiple sclerosis patients. Ann Neurol. (2011) 69:408–13. 10.1002/ana.22352 21387383

[19] Kappos L Mehling M Arroyo R Izquierdo G Selmaj K Curovic-Perisic V. Randomized trial of vaccination in fingolimod-treated patients with multiple sclerosis. Neurology. (2015) 84:872–9. 10.1212/WNL.0000000000001302 25636714

[20] Kaufman M Pardo G Rossman H Sweetser MT Forrestal F Duda P. Natalizumab treatment shows no clinically meaningful effects on immunization responses in patients with relapsing-remitting multiple sclerosis. J Neurol Sci. (2014) 341:22–7. 10.1016/j.jns.2014.03.035 24731783

[21] Bar-Or A Calkwood JC Chognot C Evershed J Fox EJ Herman A. Effect of ocrelizumab on vaccine responses in patients with multiple sclerosis: the VELOCE study. Neurology. (2020) 95:e1999–2008. 10.1212/WNL.0000000000010380 32727835 PMC7843152

[22] McCarthy CL Tuohy O Compston DA Kumararatne DS Coles AJ Jones JL. Immune competence after alemtuzumab treatment of multiple sclerosis. Neurology. (2013) 81:872–6. 10.1212/WNL.0b013e3182a35215 23925762 PMC3885219

[23] Mehta L Umans K Ozen G Robinson RR Elkins J. Immune response to seasonal influenza vaccine in patients with relapsing-remitting multiple sclerosis receiving long-term daclizumab beta: a prospective, open-label, single-arm study. Int J MS Care. (2017) 19:141–7. 10.7224/1537-2073.2016-026 28603462 PMC5460867

[24] Metze C Winkelmann A Loebermann M Hecker M Schweiger B Reisinger EC. Immunogenicity and predictors of response to a single dose trivalent seasonal influenza vaccine in multiple sclerosis patients receiving disease-modifying therapies. CNS Neurosci Ther. (2019) 25:245–54. 10.1111/cns.13034 30044050 PMC6488907

[25] Olberg HK Eide GE Cox RJ Jul-Larsen A Lartey SL Vedeler CA. Antibody response to seasonal influenza vaccination in patients with multiple sclerosis receiving immunomodulatory therapy. Eur J Neurol. (2018) 25:527–34. 10.1111/ene.13537 29205701

[26] Olberg HK Cox RJ Nostbakken JK Aarseth JH Vedeler CA Myhr KM. Immunotherapies influence the influenza vaccination response in multiple sclerosis patients: an explorative study. Mult Scler. (2014) 20:1074–80. 10.1177/1352458513513970 24436455

[27] Sormani MP Inglese M Schiavetti I Carmisciano L Laroni A Lapucci C. Effect of SARS-CoV-2 mRNA vaccination in MS patients treated with disease modifying therapies. EBioMedicine. (2021) 72:103581. 10.2139/ssrn.3886420 34563483 PMC8456129

[28] Disanto G Sacco R Bernasconi E Martinetti G Keller F Gobbi C. Association of disease-modifying treatment and anti-CD20 infusion timing with humoral response to 2 SARS-CoV-2 vaccines in patients with multiple sclerosis. JAMA Neurol. (2021) 78:1529–31. 10.1001/jamaneurol.2021.3609 34554185 PMC8461551

[29] European Agency for the Evaluation of Medicinal Products. Committee for Proprietary Medicinal Products: Note for Guidance on Harmonization of Requirements for Influenza Vaccines. London: European Agency for the Evaluation of Medicinal Products (1997).

[30] Russell MW Moldoveanu Z Ogra PL Mestecky J. Mucosal immunity in COVID-19: a neglected but critical aspect of SARS-CoV-2 infection. Front Immunol. (2020) 11:611337. 10.3389/fimmu.2020.611337 33329607 PMC7733922

[31] Lan J Ge J Yu J Shan S Zhou H Fan S. Structure of the SARS-CoV-2 spike receptor-binding domain bound to the ACE2 receptor. Nature. (2020) 581:215–20. 10.1038/s41586-020-2180-5 32225176

[32] Centonze D Rocca MA Gasperini C Kappos L Hartung HP Magyari M. Disease-modifying therapies and SARS-CoV-2 vaccination in multiple sclerosis: an expert consensus. J Neurol. (2021) 268:3961–8. 10.1007/s00415-021-10545-2 33844056 PMC8038920

[33] Fact Sheet for Healthcare Providers Administering Vaccine (Vaccination Providers): Emergency Use Authorization (EUA) of the Pfizer-BioNTech COVID-19 Vaccine to Prevent Coronavirus Disease 2019 (COVID-19). New York, NY: Pfizer Inc. (2021).

[34] Fact Sheet for Healthcare Providers Administering Vaccine (Vaccination Providers): Emergency Use Authorization (EUA) of the Moderna COVID-19 Vaccine to Prevent Coronavirus Disease 2019 (COVID-19). Cambridge, MA: Moderna TX Inc. (2021).

[35] Fact Sheet for Healthcare Providers Administering Vaccine (Vaccination Providers): Emergency Use Authorization (EUA) of the Janssen COVID-19 Vaccine to Prevent Coronavirus Disease 2019 (COVID-19). Horsham, PA: Janssen Pharmaceutical Company of Johnson & Johnson (2021).

[36] Voysey M Costa Clemens SA Madhi SA Weckx LY Folegatti PM Aley PK. Single-dose administration and the influence of the timing of the booster dose on immunogenicity and efficacy of ChAdOx1 nCoV-19 (AZD1222) vaccine: a pooled analysis of four randomised trials. Lancet. (2021) 397:881–91. 10.1016/S0140-6736(21)00432-3 33617777 PMC7894131

[37] Keech C Albert G Cho I Robertson A Reed P Neal S. Phase 1-2 trial of a SARS-CoV-2 recombinant spike protein nanoparticle vaccine. N Engl J Med. (2020) 383:2320–32. 10.1056/NEJMoa2026920 32877576 PMC7494251

[38] Logunov DY Dolzhikova IV Shcheblyakov DV Tukhvatulin AI Zubkova OV Dzharullaeva AS. Safety and efficacy of an rAd26 and rAd5 vector-based heterologous prime-boost COVID-19 vaccine: an interim analysis of a randomised controlled phase 3 trial in Russia. Lancet. (2021) 397:671–81. 10.1016/S0140-6736(21)00234-8 33545094 PMC7852454

[39] Wu Z Hu Y Xu M Chen Z Yang W Jiang Z. Safety, tolerability, and immunogenicity of an inactivated SARS-CoV-2 vaccine (CoronaVac) in healthy adults aged 60 years and older: a randomised, double-blind, placebo-controlled, phase 1/2 clinical trial. Lancet Infect Dis. (2021) 21:803–12. 10.1016/S1473-3099(20)30987-7 33548194 PMC7906628

[40] SAGE Working Group on COVID-19 Vaccines. Evidence assessment: Sinopharm/BBIBP COVID-19 Vaccine. World Health Organization (2021).

[41] Conte WL. Attenuation of antibody response to SARS-CoV-2 infection in patients with multiple sclerosis on ocrelizumab: a case-control study. Mult Scler Relat Disord. (2021) 52:103014. 10.1016/j.msard.2021.103014 34000684 PMC8102087

[42] Apostolidis SA Kakara M Painter MM Goel RR Mathew D Lenzi K. Cellular and humoral immune responses following SARS-CoV-2 mRNA vaccination in patients with multiple sclerosis on anti-CD20 therapy. Nat Med. (2021) 27:1990–2001. 10.1101/2021.06.23.21259389 34522051 PMC8604727

[43] Zabalza A Arrambide G Otero-Romero S Pappolla A Tagliana P López-Maza S. Humoral and cellular responses to SARS-CoV-2 vaccination in patients with multiple sclerosis and other autoimmune diseases. Mult Scler. (2021) 27(2_Suppl.):P945. 10.1177/13524585221089540 35475363

[44] Tortorella C Aiello A Gasperini C Agrati C Castilletti C Ruggieri S. Humoral- and T-cell–specific immune responses to SARS-CoV-2 mRNA vaccination in patients with MS using different disease-modifying therapies. Neurology. (2021). 10.1212/WNL.0000000000013108. [Epub ahead of print]. 34810244 PMC8826460

[45] Farez MF Correale J Armstrong MJ Rae-Grant A Gloss D Donley D. Practice guideline update summary: Vaccine-preventable infections and immunization in multiple sclerosis: report of the Guideline Development, Dissemination, and Implementation Subcommittee of the American Academy of Neurology. Neurology. (2019) 93:584–94. 10.1212/WNL.0000000000008157 31462584

[46] Berger JR Brandstadter R Bar-Or A. COVID-19 and MS disease-modifying therapies. Neurol Neuroimmunol Neuroinflamm. (2020) 7:761. 10.1212/NXI.0000000000000761 32414755 PMC7238896

[47] Sharifian-Dorche M Sahraian MA Fadda G Osherov M Sharifian-Dorche A Karaminia M. COVID-19 and disease-modifying therapies in patients with demyelinating diseases of the central nervous system: a systematic review. Mult Scler Relat Disord. (2021) 50:102800. 10.1016/j.msard.2021.102800 33578206 PMC7845520

[48] Mohn N Konen FF Pul R Kleinschnitz C Pruss H Witte T. Experience in multiple sclerosis patients with COVID-19 and disease-modifying therapies: a review of 873 published cases. J Clin Med. (2020) 9:4067. 10.3390/jcm9124067 33339436 PMC7766122

[49] Bar-Or A Pachner A Menguy-Vacheron F Kaplan J Wiendl H. Teriflunomide and its mechanism of action in multiple sclerosis. Drugs. (2014) 74:659–74. 10.1007/s40265-014-0212-x 24740824 PMC4003395

[50] Bollo L Guerra T Bavaro DF Monno L Saracino A Angarano G. Seroconversion and indolent course of COVID-19 in patients with multiple sclerosis treated with fingolimod and teriflunomide. J Neurol Sci. (2020) 416:117011. 10.1016/j.jns.2020.117011 32650143 PMC7339938

[51] Ciardi MR Zingaropoli MA Pasculli P Perri V Tartaglia M Valeri S. The peripheral blood immune cell profile in a teriflunomide-treated multiple sclerosis patient with COVID-19 pneumonia. J Neuroimmunol. (2020) 346:577323. 10.1016/j.jneuroim.2020.577323 32688146 PMC7361089

[52] Laroni A Schiavetti I Sormani MP Uccelli A. COVID-19 in patients with multiple sclerosis undergoing disease-modifying treatments. Mult Scler. (2020) 18:1352458520971817. 10.1177/1352458520971817 33205695

